# Nontherapeutic Risk Factors of Different Grouped Stage IIIC Breast Cancer Patients' Mortality: A Study of the US Surveillance, Epidemiology, and End Results Database

**DOI:** 10.1155/2022/6705052

**Published:** 2022-08-30

**Authors:** Yue Qiu, Hongye Chen, Yongjing Dai, Baoshi Bao, Lin Tian, Yuhui Chen

**Affiliations:** ^1^Chinese PLA Medical School, Beijing 100853, China; ^2^Department of General Surgery, The First Medical Center, Chinese PLA General Hospital, Beijing 100853, China

## Abstract

**Objectives:**

Stage IIIC breast cancer, as a local advanced breast cancer, has a poor prognosis compared with that of early breast cancer. We further investigated the risk factors of mortality in stage IIIC primary breast cancer patients and their predictive value.

**Methods:**

We extracted data from the US Surveillance, Epidemiology, and End Results (SEER) database of female patients with stage IIIC primary breast cancer (*n* = 1673) from January 2011 to December 2015.

**Results:**

Hormone receptor negativity (*P* ≤ 0.001 and *P* ≤ 0.001, respectively), aggressive molecular typing (*P* ≤ 0.001 and *P* ≤ 0.001, respectively), high *T* stage (*P* ≤ 0.001 and *P* ≤ 0.001, respectively), a high number of positive lymph nodes (≥14) (*P*=0.005 and *P*=0.001, respectively), and lymph node ratio (≥0.8148) (*P* ≤ 0.001 and *P* ≤ 0.001, respectively) were associated with poor disease-specific survival. The indicators of disease-specific survival included estrogen receptor status, progesterone receptor status, molecular typing, *T* stage, number of positive lymph nodes, and lymph node ratio (*P* ≤ 0.001,*P* ≤ 0.001,*P* ≤ 0.001,*P* ≤ 0.001, *P*=0.002, and *P* ≤ 0.001, respectively).

**Conclusion:**

Hormone receptor negativity, aggressive molecular typing, high *T* stage, high number of positive lymph nodes, and lymph node ratio are poor prognostic factors patients with stage IIIC primary breast cancer. The efficient indicators of disease-specific survival include estrogen receptor status, progesterone receptor status, molecular typing, *T* stage, number of positive lymph nodes, and lymph node ratio.

## 1. Introduction

In 2020, the number of new cases of breast cancer worldwide reached 2.26 million, surpassing lung cancer to rank the first in the world [1]. The prognosis of breast cancer is relatively good, and the 5-year survival rate of some breast cancer can reach as high as 99% [2]. However, studies on the staging system showed that the survival rate of breast cancer decreased with the increasing stage [3]. The 7th version of the American Joint Committee on Cancer (AJCC) TNM staging system [4] defines stage IIIC (any TN3M0) breast cancer as N3 stage breast cancer without any distant metastasis. Patients who meet one of the following conditions are diagnosed as pN3: 10 or more axillary lymph nodes metastases; subclavian lymph nodes metastases; clinically found internal mammary lymph nodes metastases with one or more axillary lymph nodes metastases; more than 3 axillary lymph nodes metastases, accompanied by clinically undetected internal mammary lymph nodes metastases confirmed by sentinel lymph node biopsy; and ipsilateral supraclavicular lymph nodes metastases. According to the aforementioned criteria, patients can be further divided into N3a, N3b, and N3c.

The 5-year overall survival (OS) and 5-year disease-specific survival (DSS) of stage IIIC breast cancer are about 61.7% and 66.8%, respectively, and its prognosis is relatively worse than that of early breast cancer [5]. Some studies have discussed the prognostic indicators of stage IIIC breast cancer, such as molecular typing and lymph node ratio (LNR). However, most of the patients were from a single institution and the factors involved were not comprehensive; so, they could not be further grouped [6–8]. Based on the US Surveillance, Epidemiology, and End Results (SEER) database, we studied the risk factors of breast cancer-specific mortality (BCSM) in patients with stage IIIC breast cancer and explored their predictive value at different levels.

## 2. Materials and Methods

### 2.1. Patients

The data supporting this study were obtained from the SEER registry. Since 1975, the SEER program has continuously collected data on cancer incidence and mortality in the United States, which is based on 9 population-based registries. We acquired the required data from the SEER database through SEER ^*∗*^ Stat software of version 8.3.9.2.

**Figure 1:**
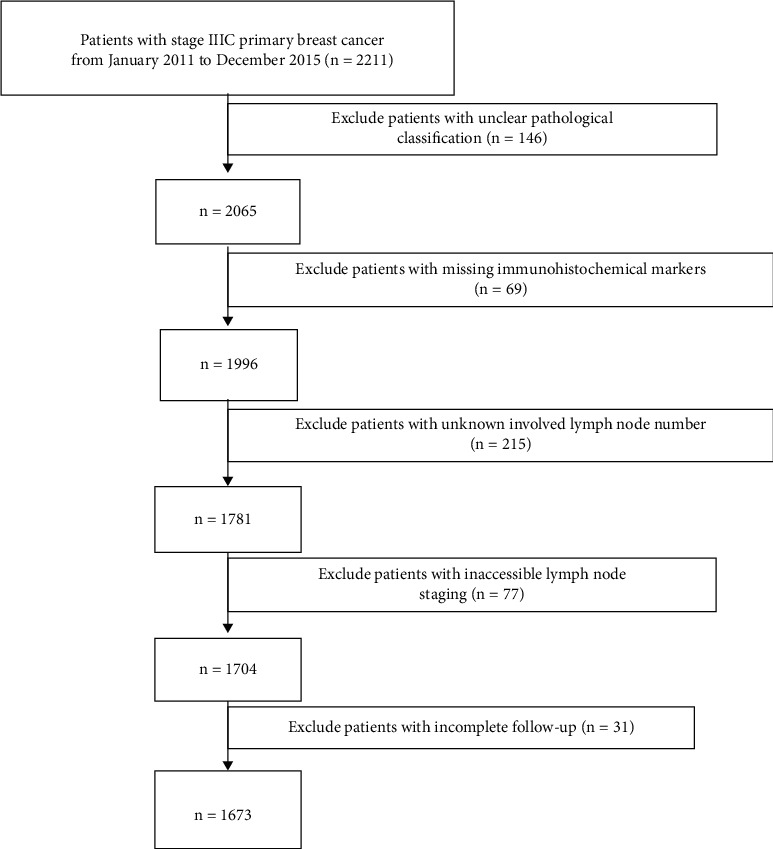
Process of data obtained from the US surveillance, Epidemiology, and End Results database.

In the client-server mode of SEER ^*∗*^Stat, we used the International Classification of Diseases for Oncology, the 3rd edition of histology, and behavior codes (ICD-O-3 morphology codes 8500–8599) to identify patients. We selected 2211 female patients diagnosed with stage IIIC primary breast cancer from January 2011 to December 2015, excluding those with unclear pathological classification (*n* = 146), missing immunohistochemical markers (*n* = 69), unknown involved lymph node number (*n* = 215), inaccessible lymph node staging (*n* = 77), and incomplete follow-up (*n* = 31). Finally, a total of 1673 patients were left (Figure 1).

### 2.2. Study Variables

We evaluated the clinicopathological features of each patient, including age at diagnosis, laterality, primary site, histological type, histological grade, molecular typing, estrogen receptor (ER) status, progesterone receptor (PR) status, human epidermal growth factor receptor 2 (HER-2) status, *T* stage, N3 stage, number of positive lymph nodes (LNP), LNR, and survival time. The age at diagnosis of patients was classified as 40 years. The LNR was defined as the ratio of the number of LNP to the total number of lymph nodes. The cutoff values of LNP and LNR, calculated by the receiver operating characteristic curve (ROC), were 14 and 0.8148, respectively.

### 2.3. Statistical Methods

All statistical analyses of this study were completed using Stata Statistical Software version 15.1 (College Station, TX: StataCorp LLC). The measurement data were described by median (interquartile range). Frequency was used to show the counting data. The Kaplan–Meier method was used in survival analysis, and the log-rank test was used to compare different survival curves. Univariate and multivariate analyses were performed using the Cox model. Finally, the ROC analysis was conducted to illustrate the discriminating ability of risk factors in terms of BCSM and report the area under the curve (AUC). Statistical significance was set as two-sided *P* < 0.05, and all confidence intervals (CI) were expressed at the 95% confidence level.

## 3. Results

### 3.1. Clinicopathological Features

Most patients with stage IIIC breast cancer were over 40 years of age (91.15% vs 8.85%). As for the histological type, patients with invasive ductal carcinoma predominated (64.02% vs 35.98%). In terms of molecular typing, luminal A patients ranked first, followed by triple negative patients (68.02% and 12.91%, respectively). The number of ER positive patients, PR positive patients, and HER-2 negative patients were far higher than their counterparts (78.00%, 65.03%, and 80.93%, respectively). The proportions of T2 stage patients and N3a stage patients were the highest (45.13% and 85.24%, respectively). There were more patients with an LNP higher than 14 and patients with an LNR higher than 0.8148 (56.37% and 55.29%) (Table 1). The median follow-up time was 54 (38∼72) months. At the end of the follow-up, 578 patients died, of whom 447 died of breast cancer. The 5-year OS and DSS were 51.72% (95% CI: 0.4838∼0.5495) and 60.74% (95% CI: 0.5735∼0.6395), respectively.

### 3.2. Univariate Analysis Related to DSS

Univariate analysis showed that poor molecular typing, ER negativity, PR negativity, HER-2 negativity, high *T* stage, and high N3 stage were associated with poor DSS (*P* ≤ 0.001, *P* ≤ 0.001, *P* ≤ 0.001, *P*=0.017, *P* ≤ 0.001, and *P* ≤ 0.001, respectively). A high number of LNP (≥14) and LNR (≥0.8148) were associated with poor DSS (*P*=0.001 and *P* ≤ 0.001, respectively) (Table 2).

### 3.3. Multivariate Analysis by the Cox Model Assessing Factors Associated with DSS

In multivariate analysis, ER negativity, PR negativity, aggressive molecular typing, and high *T* stage were still associated with poor DSS (*P* ≤ 0.001,*P* ≤ 0.001, *P* ≤ 0.001, and *P* ≤ 0.001, respectively). A higher number of LNP (≥14) and LNR (≥0.8148) were also associated with poor DSS (*P*=0.005 and *P* ≤ 0.001, respectively). There was a borderline level of statistical significance between the N3c stage and DSS (Hazard ratio: 1.419, 95% CI: 0.982∼2.051,*P*=0.063) (Table 3).

### 3.4. Survival Analysis

**Figure 2:**
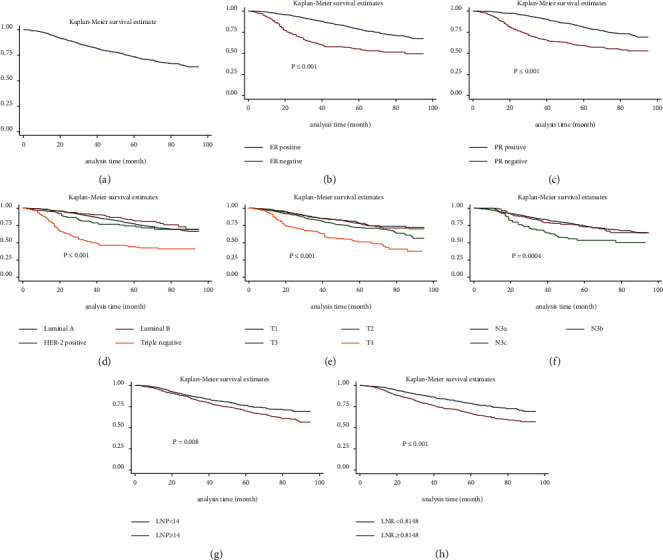
Kaplan–Meier analysis of DSS. (a). Kaplan–Meier estimates of DSS for patients with stage IIIC breast cancer. (b). Kaplan–Meier analysis comparing DSS in patients with different ER statuses. (c). Kaplan–Meier analysis comparing DSS in patients with different PR statuses. (d). Kaplan–Meier analysis comparing DSS in patients with different molecular typing. (e). Kaplan–Meier analysis comparing DSS in patients with different T stages. (f). Kaplan–Meier analysis comparing DSS in patients with different N3 stages. (g). Kaplan–Meier analysis comparing DSS in patients with different LNP. (h). Kaplan–Meier analysis comparing DSS in patients with different LNR. DSS: disease-specific survival. ER: estrogen receptor. LNP: positive lymph nodes. LNR: lymph node ratio. PR: progesterone receptor.

The 5-year OS and DSS were 51.72% (95% CI: 0.4838∼0.5495) and 60.74% (95% CI: 0.5735∼0.6395), respectively. The DSS curve is displayed in Figure 2(a). The survival rate of patients with hormone receptor (HR) positivity was significantly higher (*P* ≤ 0.001 and *P* ≤ 0.001, respectively; Figures 2(b) and 2(c)). The prognosis of patients with an aggressive molecular typing was poor (*P* ≤ 0.001, Figure 2(d)). Moreover, the higher the *T* stage and N3 stage were, the lower the survival rate of patients was (*P* ≤ 0.001 and *P*=0.0004, respectively; Figures 2(e) and 2(f)). We also found that patients with LNP <14 and LNR <0.8148 had a better prognosis (*P*=0.0008 and *P* ≤ 0.001, respectively; Figures 2(g) and 2(h)).

### 3.5. Predictive Value of Risk Factors

**Figure 3:**
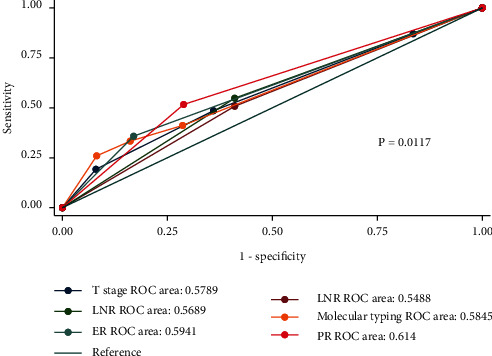
The ROC curve and AUC showing the discrimination ability of the ER status, PR status, molecular typing, T stage, LNP number, and LNR in stage IIIC breast cancer. AUC: area under the curve. ER: estrogen receptor. LNP: positive lymph nodes. LNR: lymph node ratio. PR: progesterone receptor. ROC: receiver operating characteristic curve.

**Figure 4:**
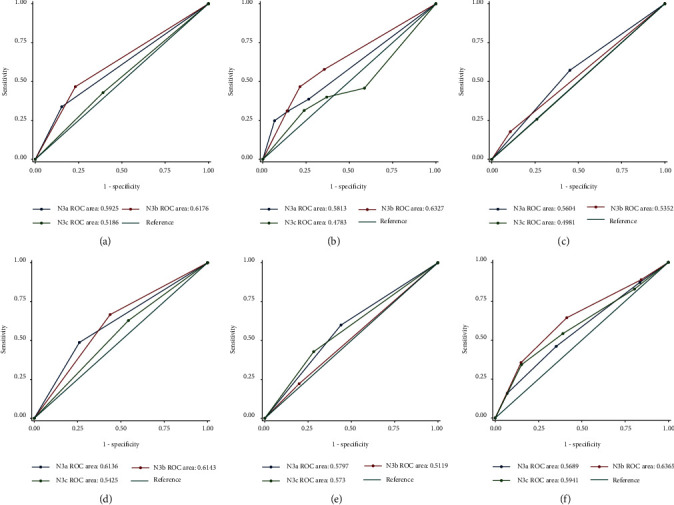
The ROC curve and AUC of risk factors of DSS in patients with different N3 stages. (a). Comparison of the ROC curve and AUC of the ER status in patients with different N3 stages. (b). Comparison of the ROC curve and AUC of molecular typing in patients with different N3 stages. (c). Comparison of the ROC curve and AUC of LNP in patients with different N3 stages. (d). Comparison of the ROC curve and AUC of the PR status in patients with different N3 stages. (e). Comparison of the ROC curve and AUC of LNR in patients with different N3 stages. (f). Comparison of the ROC curve and AUC of the T stage in patients with different N3 stages. AUC: area under the curve. DSS: disease-specific survival. ER: estrogen receptor. LNP: positive lymph nodes. LNR: lymph node ratio. PR: progesterone receptor. ROC: receiver operating characteristic curve.

The ROC curve and AUC showed the discrimination ability of the ER status, PR status, *T* stage, molecular typing, LNP number, and LNR in stage IIIC breast cancer (Figure 3). The indicators for predicting DSS in patients with stage IIIC breast cancer included ER status, PR status, molecular typing, *T* stage, LNP number, and LNR (*P* ≤ 0.001, *P* ≤ 0.001, *P* ≤ 0.001, *P* ≤ 0.001, *P*=0.002, and *P* ≤ 0.001, respectively; Table 4). At the same time, we reported the ROC curve and AUC of the abovementioned predictors in patients with different N3 stages (Table 5 and Figure 4). The results showed that the predictors of DSS in patients with N3a stage were ER status, PR status, molecular typing, *T* stage, LNP number, and LNR (*P* ≤ 0.001, *P* ≤ 0.001, *P* ≤ 0.001, *P* ≤ 0.001, *P* ≤ 0.001, and *P* ≤ 0.001, respectively). The indicators with a good predictive value for DSS in patients with N3b stage only included the ER status, PR status, molecular typing, and *T* stage (*P*=0.020, *P*=0.024, *P*=0.009, and *P*=0.007, respectively); however, these indicators had a poor predictive ability for DSS in N3c patients. Meanwhile, there was no significant difference in the DSS prediction ability of each index in patients with different N3 stages (*P* > 0.05).

## 4. Discussion

The 5-year OS of stage IIIC breast cancer was about 61%∼73%, and it was reported that the prognosis was not good [9, 10]. The median follow-up time of patients in this study was 54 (38∼72) months, the restricted mean DSS was 76.027 (95% CI: 74.5258∼77.5287) months, and the 5-year OS and 5-year DSS were 51.72% (95% CI: 0.4838∼0.5495) and 60.74% (95% CI: 0.5735∼6395), respectively. However, not all patients with stage IIIC breast cancer have the same clinicopathological characteristics and prognosis. For example, some researchers believed that the risk of BCSM in HR-negative stage IIIC breast cancer patients in 20 years was significantly higher than that in HR-positive patients (68.7% vs 63.1%) [11].

At the beginning, we discussed the clinicopathological features of patients with stage IIIC breast cancer and found that stage IIIC breast cancer patients had old age, low HER-2 positivity rate, and a high HR positivity rate. In terms of molecular typing, Luminal A patients ranked first, followed by triple negative patients (68.02% and 12.91%, respectively). The HR and HER-2 status are recognized prognostic markers in breast cancer. Compared with other subtypes, the overall prognosis of triple negative breast cancer was poor [12, 13], while the overall prognosis of Luminal B breast cancer was the best [14, 15]. Our Kaplan–Meier analysis also confirmed this point. The survival rates of HR-positive patients were higher than those of HR-negative patients (*P* ≤ 0.001). As for the histological type, patients with invasive ductal carcinoma predominated (64.02% vs 35.98%). After matching, invasive ductal carcinoma had similar OS to invasive lobular carcinoma, but invasive lobular carcinoma patients with risk factors had worse outcomes [16]. In addition, a high proportion of LNP <14 and LNR <0.8148 were also the characteristics of these patients. Notably, after breast cancer patients with supraclavicular nodal metastases downstaged from AJCC stage IV to IIIC, N3c patients who received the standard therapy demonstrated better OS than stage IV disease [17, 18]. Hence, we sought to illustrate whether there was a difference between stage IIIC patients.

In this study, univariate analysis showed that poor molecular typing, ER negativity, PR negativity, HER-2 negativity, high *T* stage, and high N3 stage were associated with poor DSS (*P* ≤ 0.001, *P* ≤ 0.001, *P* ≤ 0.001, *P*=0.017, *P* ≤ 0.001 and *P* ≤ 0.001, respectively). In addition, the prognosis of patients with stage IIIC breast cancer varied with different N3 stages. Real-world studies found that patients with the N3c stage had the worst prognosis, followed by N3a patients, while N3b patients had the best prognosis. Both univariate and multivariate analyses showed that HR negativity (*P* ≤ 0.001 and *P* ≤ 0.001, respectively), aggressive molecular typing (*P* ≤ 0.001 and *P* ≤ 0.001, respectively), high *T* stage (*P* ≤ 0.001 and *P* ≤ 0.001, respectively), a high number of LNP (≥14) (*P*=0.001 and *P*=0.005, respectively), and LNR (≥0.8148) (*P* ≤ 0.001, and *P* ≤ 0.001, respectively) were associated with poor DSS. When the 7th version of the AJCC TNM staging system took N staging and the number of LNP as lymph node-related prognostic indicators, some researchers had questioned whether it was the most accurate way to represent the state of metastatic cancer in lymph nodes and proposed that LNR should be considered to be more valuable [19, 20]. In patients with stage II to III breast cancer, LNR may be a more useful tool for predicting survival than pathological lymph node staging [21–23].

We drew the ROC curve of the abovementioned prediction indicators and reported the AUC. The predictors of DSS for patients with stage IIIC breast cancer included ER status, PR status, molecular typing, *T* stage, LNP number, and LNR (*P* ≤ 0.001, *P* ≤ 0.001, *P* ≤ 0.001, *P* ≤ 0.001, *P*=0.002, and *P* ≤ 0.001, respectively). In some studies, large tumor size, lymph node metastasis, and HR negativity were often considered to be adverse prognostic factors [24–26]. In multivariate analysis, older age at diagnosis, higher N stage, and ER negativity were found to be independent predictors of BCSM [27]. We wanted to know whether the predictive value of these indicators was different from patients with different N3 stages. Therefore, patients were further grouped according to the N3 stage and we found that the indexes with predictive value for DSS of N3a patients were ER status, PR status, molecular typing, *T* stage, LNP number, and LNR (*P* ≤ 0.001, *P* ≤ 0.001, *P* ≤ 0.001, *P* ≤ 0.001, and *P* ≤ 0.001, respectively). The indicators with a good predictive value for DSS in patients with the N3b stage only included ER status, PR status molecular typing, and *T* stage (*P*=0.020 , *P*=0.024, *P*=0.009, and *P*=0.007, respectively). However, these indicators had a poor predictive ability in N3c patients. In previous studies, grade III disease, perineural invasion, LNP ≥20, and LNR ≥0.9 were found to be associated with the OS and disease-free survival of N3a stage breast cancer [28, 29]. For N3b stage breast cancer, pathological prognostic staging was an independent predictor related to OS and DSS [30]. In addition, there was no significant difference in the discrimination ability of DSS among patients with different N3 stages in our study (*P* > 0.05).

Inevitably, our research also has some limitations. For example, due to the lack of detailed follow-up data, we have not discussed the effect of different treatment options on the survival of patients with stage IIIC breast cancer under modern treatment modalities. In addition, we failed to establish an effective prognosis prediction model. We still lack specific exploration and discussion on the predictive value of LNR.

## 5. Conclusion

The survival rate of HR-positive patients with stage IIIC breast cancer is significantly higher. The higher the *T* stage and N3 stage are, the lower the survival rate is. The prognosis of patients with aggressive molecular typing, LNP ≥14, and LNR ≥0.8148 is poor. HR negativity, poor molecular typing, high *T* stage and N3 stage, and high LNP and LNR are the adverse prognostic factors of stage IIIC breast cancer. The ideal predictors of BCSM in patients with stage IIIC breast cancer include ER status, PR status, molecular typing, *T* stage, LNP number, and LNR. Although the discrimination ability of the abovementioned indicators in patients with different N3 stages is dissimilar, there is no statistical difference.

## Figures and Tables

**Table 1:** Clinicopathological features of patients with stage IIIC breast cancers.

Category	*n* = 1673
*Age at diagnosis (year)*	
≤40 years old	148 (8.85%)
>40 years old	1525 (91.15%)

*Laterality*	
Left	832 (49.73%)
Right	841 (50.27%)

*Primary site*	
Upper-inner	102 (6.10%)
Upper-outer	594 (35.51%)
Lower-inner	51 (3.05%)
Lower-outer	102 (6.10%)
Other types	824 (49.25%)

*Histological type*	
Infiltrating duct carcinoma	1071 (64.02%)
Lobular carcinoma	354 (21.16%)
Infiltrating duct and lobularcarcinoma	177 (10.58%)
Other types	71 (4.24%)

*Histological grade*	
Well-differentiated	149 (8.91%)
Moderately differentiated	748 (44.71%)
Poorly differentiated	772 (46.14%)
Undifferentiated	4 (0.24%)

*Molecular typing*	
Luminal A	1138 (68.02%)
Luminal B	188 (11.24%)
HER-2 positive	131 (7.83%)
Triple negative	216 (12.91%)

*ER status*	
Positive	1305 (78.00%)
Negative	368 (22.00%)

*PR status*	
Positive	1088 (65.03%)
Negative	585 (34.97%)

*HER-2 status*	
Positive	319 (19.07%)
Negative	1354 (80.93%)

*T stage*	
T1	260 (15.54%)
T2	755 (45.13%)
T3	474 (28.33%)
T4	184 (11.00%)

*N3 stage*	
N3a	1426 (85.24%)
N3b	166 (9.92%)
N3c	81 (4.84%)

*LNP*	
<14	943 (56.37%)
≥14	730 (43.63%)

*LNR*	
<0.8148	925 (55.29%)
≥0.8148	748 (44.71%)

Follow-up time (month), IQR	54 (38∼72)
5-year OS	51.72% (95% CI: 0.4838∼0.5495)
5-year DSS	60.74% (95% CI: 0.5735∼0.6395)

ER: estrogen receptor. HER-2: human epidermal growth factor receptor 2. LNP: positive lymph nodes. LNR: lymph node ratio. PR: progesterone receptor. IQR: interquartile range. DSS: disease-specific survival. OS: overall survival. CI: confidence interval.

**Table 2:** Univariate analysis related to DSS.

Variable	HR (95% CI)	*P* value
*Age at diagnosis (year)*		
≤40 years old	1	
>40 years old	1.121 (0.824∼1.526)	0.467

*Laterality*		
Left	1	
Right	1.020 (0.848∼1.228)	0.832

*Primary site*		
Upper-inner	1	
Upper-outer	1.009 (0.677∼1.505)	0.964
Lower-inner	0.662 (0.329∼1.329)	0.246
Lower-outer	0.921 (0.543∼1.563)	0.761
Other types	0.833 (0.562∼1.236)	0.365

*Histological type*		
Infiltrating duct carcinoma	1	
Lobular carcinoma	0.994 (0.786∼1.258)	0.962
Infiltrating duct and lobularcarcinoma	1.251 (0.933∼1.678)	0.134
Other types	1.099 (0.699∼1.731)	0.681

*Histological grade*		
Well-differentiated	1	
Moderately differentiated	0.884 (0.631∼1.240)	0.477
Poorly differentiated	1.021 (0.731∼1.425)	0.904
Undifferentiated ^*∗*^	—	—

*Molecular typing*		
Luminal A	1	
Luminal B	0.775 (0.544∼1.102)	0.156
HER-2 positive	1.206 (0.840∼1.732)	0.310
Triple negative	3.617 (2.904∼4.505)	≤0.001

*ER status*		
Positive	1	
Negative	2.661 (2.192∼3.230)	≤0.001

*PR status*		
Positive	1	
Negative	2.573 (2.137∼3.099)	≤0.001

*HER-2 status*		
Positive	1	
Negative	1.368 (1.057∼1.771)	0.017

*T stage*		
T1	1	
T2	1.068 (0.793∼1.438)	0.666
T3	1.402 (1.029∼1.910)	0.032
T4	3.011 (2.157∼4.204)	≤0.001

*N3 stage*		
N3a	1	
N3b	1.076 (0.789∼1.466)	0.644
N3c	1.975 (1.396∼2.795)	≤0.001

*LNP*		
<14	1	
≥14	1.372 (1.140∼1.652)	0.001

*LNR*		
<0.8148	1	
≥0.8148	1.682 (1.396∼2.026)	≤0.001

^*∗*^Among the patients who died of breast cancer included in this study, there were no patients belonging to this category. CI: confidence interval. DSS: disease-specific survival. ER: estrogen receptor. HER-2: human epidermal growth factor receptor 2. HR: hazard ratio. LNP: positive lymph nodes. LNR: lymph node ratio. PR: progesterone receptor.

**Table 3:** Multivariate analysis by the cox model assessing factors associated with DSS.

Variable	HR (95% CI)	*P* value
*Age at diagnosis (year)*		
≤40 years old	1	
>40 years old	1.210 (0.881∼1.661)	0.238

*Laterality*		
Left	1	
Right	1.060 (0.878∼1.280)	0.545

*Primary site*		
Upper-inner	1	
Upper-outer	1.016 (0.678∼1.523)	0.938
Lower-inner	0.748 (0.369∼1.514)	0.420
Lower-outer	0.788 (0.460∼1.351)	0.387
Other types	0.871 (0.584∼1.299)	0.498

*Histological type*		
Infiltrating duct carcinoma	1	
Lobular carcinoma	1.115 (0.859∼1.449)	0.413
Infiltrating duct and lobularcarcinoma	1.192 (0.878∼1.618)	0.259
Other types	1.251 (0.791∼1.976)	0.338

*Histological grade*		
Well-differentiated	1	
Moderately differentiated	1.005 (0.710∼1.421)	0.979
Poorly differentiated	1.194 (0.832∼1.71)	0.336
Undifferentiated ^*∗*^	—	—

*Molecular typing*		
Luminal A	1	
Luminal B	0.588 (0.408∼0.848)	0.004
HER-2 positive	0.168 (0.079∼0.359)	≤0.001
Triple negative	0.491 (0.243∼0.991)	0.047

*ER status*		
Positive	1	
Negative	3.632 (1.939∼6.804)	≤0.001

*PR status*		
Positive	1	
Negative	2.276 (1.751∼2.960)	≤0.001

*HER-2 status*		
Positive	1	
Negative	1	—

*T stage*		
T1	1	
T2	1.112 (0.823∼1.503)	0.489
T3	1.473 (1.077∼2.013)	0.015
T4	2.950 (2.093∼4.158)	≤0.001

*N3 stage*		
N3a	1	
N3b	0.967 (0.693∼1.349)	0.844
N3c	1.419 (0.982∼2.051)	0.063

*LNP*		
<14	1	
≥14	1.350 (1.097∼1.662)	0.005

*LNR*		
<0.8148	1	
≥0.8148	1.495 (1.221∼1.831)	≤0.001

^*∗*^Among the patients who died of breast cancer included in this study, there were no patients belonging to this category. CI: confidence interval. DSS: disease-specific survival. ER: estrogen receptor. HER-2: human epidermal growth factor receptor 2. HR: hazard ratio. LNP: positive lymph nodes. LNR: lymph node ratio. PR: progesterone receptor.

**Table 4:** Predictive value of risk factors of DSS.

Factor	AUC (95% CI)	*P* value
ER status	0.5941 (0.570∼0.619)	≤0.001
PR status	0.6140 (0.588∼0.640)	≤0.001
Molecular typing	0.5845 (0.557∼0.612)	≤0.001
T stage	0.5789 (0.549∼0.609)	≤0.001
LNP	0.5488 (0.522∼0.576)	0.002
LNR	0.5689 (0.542∼0.596)	≤0.001

AUC: area under the curve. CI: confidence interval. DSS: disease-specific survival. ER: estrogen receptor. LNP: positive lymph nodes. LNR: lymph node ratio. PR: progesterone receptor.

**Table 5:** Predictors of DSS in patients with different N3 stages.

Factor	N3a (*n* = 1426) AUC (95% CI)	*P*	N3b (*n* = 166) AUC (95% CI)	*P*	N3c (*n* = 81) AUC (95% CI)	*P*	*χ * ^2^	*P* value
ER status	0.5925 (0.566∼0.619)	≤0.001	0.6176 (0.535∼0.700)	0.020	0.5186 (0.409∼0.628)	0.775	2.09	0.3525
PR status	0.6136 (0.585∼0.642)	≤0.001	0.6143 (0.532∼0.697)	0.024	0.5425 (0.434∼0.652)	0.514	1.54	0.4636
Molecular typing	0.5813 (0.551∼0.612)	≤0.001	0.6327 (0.542∼0.723)	0.009	0.4783 (0.355∼0.602)	0.745	3.90	0.1420
T stage	0.5689 (0.536∼0.602)	≤0.001	0.6365 (0.541∼0.732)	0.007	0.5941 (0.470∼0.718)	0.149	1.79	0.4088
LNP	0.5604 (0.531∼0.590)	≤0.001	0.5352 (0.472∼0.598)	0.495	0.4981 (0.401∼0.596)	0.977	1.76	0.4146
LNR	0.5797 (0.551∼0.609)	≤0.001	0.5119 (0.441∼0.583)	0.814	0.5730 (0.467∼0.679)	0.260	2.99	0.2238

AUC: area under the curve. CI: confidence interval. DSS: disease-specific survival. ER: estrogen receptor. LNP: positive lymph nodes. LNR: lymph node ratio. PR: progesterone receptor.

## Data Availability

All data generated or analyzed during this study are included in this article.
